# Assessment of medical professionalism using the Professionalism Mini Evaluation Exercise (P-MEX) in a multi-ethnic society: a Delphi study

**DOI:** 10.1186/s12909-020-02147-9

**Published:** 2020-07-14

**Authors:** Warren Fong, Yu Heng Kwan, Sungwon Yoon, Jie Kie Phang, Julian Thumboo, Ying Ying Leung, Swee Cheng Ng

**Affiliations:** 1 Department of Rheumatology and Immunology, Singapore General Hospital, 20 College Road, Singapore, 169856 Singapore; 2 Duke-NUS Medical School, Singapore, Singapore; 3 NUS Yong Loo Lin School of Medicine, National University of Singapore, Singapore, Singapore; 4 Program in Health Services and Systems Research, Duke-NUS Medical School, Singapore, Singapore

**Keywords:** Professionalism, Singapore, Delphi, Assessment

## Abstract

**Background:**

The importance of medical professionalism and its assessment has been well documented in the literature. However, there is currently no culturally-adapted tool to assess medical professionalism in Singapore. This study sets out to find consensus on relevance of the items from the Professionalism Mini Evaluation Exercise (P-MEX) for assessing medical professionalism in Singapore.

**Methods:**

A two-round Delphi survey was completed by an expert panel consisting of program directors, associate designated institutional officials, and designated institutional official (*n* = 15) from residency programs in Singapore. Round 1 comprised of 23 items from the P-MEX rated on a 5-point scale (1 = Definitely include, 2 = Possibly include, 3 = Neutral, 4 = Possibly exclude, 5 = Definitely exclude). In round 2, the experts received feedback from the previous round, and were asked to re-rate the items which did not achieve consensus in the previous round. The threshold for consensus in the study was defined as 70% or greater agreement among experts.

**Results:**

Completed questionnaires for both rounds were received from all 15 experts. In round 1, 18 items (78%) achieved consensus to be included. In round 2, 1 (4%) item achieved consensus to be included. However, none of the remaining items achieved consensus to be removed, and they exhibited stability in responses. A list of 19 items covering four domains of medical professionalism (Doctor-patient relationship skills, Reflective skills, Time management and Inter-professional relationship skills) was obtained during the two-rounds of Delphi survey.

**Conclusions:**

Nineteen items in the P-MEX had garnered consensus and is suitable for assessing medical professionalism in Singapore. The findings of this study can provide guidance for faculty and institutions who want to assess medical professionalism of their medical trainees.

## Introduction

Medical professionalism has been shown to affect doctors’ relationships with their patients, quality of care, and ultimately health and illness outcomes [1]. In recent years, medical professionalism has been increasingly emphasized in medical undergraduate and post-graduate curricula [2–5]. Previously ‘good medical practice’ has been defined more broadly in terms of roles such as ‘professional’ and ‘healer’, with various medical councils, academic and professional bodies having produced clear documentations on these roles [6–8]. For residents in training, unprofessional behaviour during their training correlates with an increased risk of disciplinary action later on in their careers as physicians [9]. Fortunately, medical professionalism can be nurtured [10], but in order for this to take place, it has to be adequately assessed [11]. One of the dominant frameworks of medical professionalism is that professionalism consists of a set of behaviours and competencies that can be mastered by the physician; and these behaviours and competencies can be assessed [12]. We had previously performed a systematic review of the quality and utility of observer-based assessment tools that could be used in residency programs and had identified the Professionalism Mini-Evaluation Exercise (P-MEX) as one of the assessment tools that could be useful in the assessment of medical professionalism in our residency programs [13]. However, Chandratilake et al. and Jha et al. have demonstrated differences in understanding of professionalism in physicians from different regions [14, 15]. Moreover, according to consensus statement and recommendations from the Ottawa conference, the assessment of professionalism varies across different cultures and hence cross-cultural validation of the assessment tool is important [16]. The P-MEX was originally developed in Canada by Cruess et al [17], and when the P-MEX was piloted in Japan [18] and Finland [19], additional culturally-relevant items were added.

Since the Ottawa report in 2011 [20], studies have explored the assessment of medical professionalism in various non-Anglo-Saxon/ Western contexts such as Korea, Japan and China [21, 22]. However, till date there has not been a study looking at the assessment of medical professionalism in a multi-ethnic Asian context such as Singapore. Singapore’s multi-ethnic landscape comprises of predominantly Chinese (74%), followed by Malay (13%), and Indian (9%) [23]. The importance of cultural perspectives and its effect on medical professionalism has previously been highlighted by Jha et al [15]. The intercultural development continuum [24] and cultural fit theory [25] both highlight that professional behaviour between healthcare professionals can potential be influenced by the shared values and societal culture, and this is because professional behaviours and values are socially constructed [26]. Based on Hofstede’s cultural dimension theory, national culture consists of 6 dimensions: power distance, individualism versus collectivism, masculinity versus femininity, uncertainty avoidance, and long-term orientation versus short term normative orientation [27]. Even among Asian countries, significant cultural difference across these dimensions may exist [28].

Our previous qualitative study with patients (healthcare recipients) and faculty (medical educators) had identified 23 items to assess medical professionalism, covering four domains of medical professionalism (Doctor-patient relationship skills, Reflective skills, Time management and Inter-professional relationship skills) [29]*.* The aim of this study was to gain consensus among the stakeholders in residency programs on the items to be used to assess medical professionalism in a multi-ethnic and multi-cultural Asian context. These items can form the basis for an assessment tool for the assessment of medical professionalism within residency programs.

## Methods

### Design

A modified Delphi technique with two iterative rounds was employed. To ensure strong retention of expert involvement, an upper limit of two rounds of investigation was set in this study [8]. It is also acknowledged that having a planned number of rounds is an indicator of good quality in designing a Delphi study [13].

### Participants

SingHealth is Singapore’s largest group of public healthcare institutions, consisting of four public hospitals, five national specialty centres and a network of community hospitals and polyclinics [30]. There are over 1600 faculty and more than 900 residents in training.

Fifteen experts from the SingHealth residency programs were selected according to age, gender, ethnicity and disciplines. The experts selected had at least 3 years of experience in the residency program. They also had to be in involved in the disciplinary committees set up to investigate disciplinary issues related to residents, as well as be involved in the training and assessment of residents, especially in the area of medical professionalism. Each potential expert was sent an invitation email introducing the study objectives and the study procedures. Depending on the expert’s preference, either hardcopy or electronic questionnaire was provided upon agreement to participate in the study.

### Round 1

**Fig. 1:**
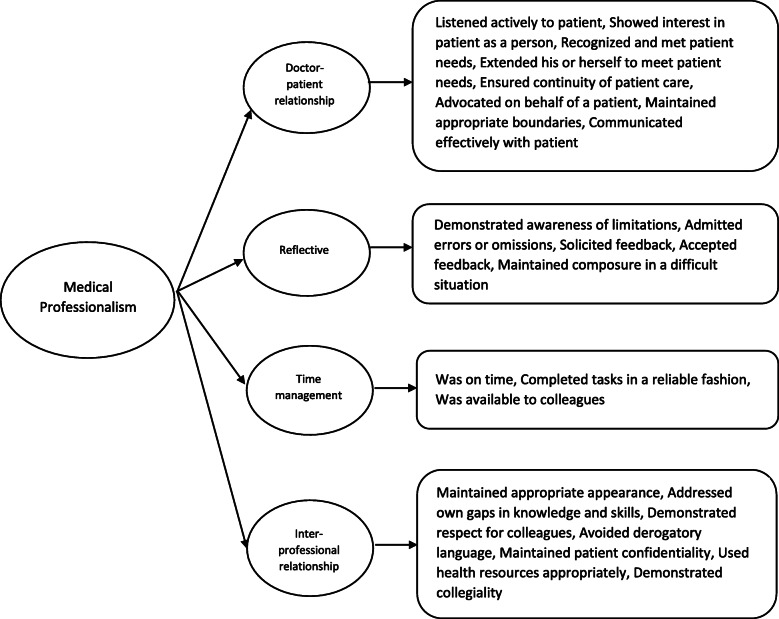
Domains and subdomains of medical professionalism

The first questionnaire contained 23 observer-based items assessing medical professionalism based on the results of the previous survey and qualitative study (Fig. 1) [29]. Two new items which emerged from the qualitative study [30]-(1) communicated effectively with patient and (2) demonstrated collegiality were included in this Delphi survey in addition to the original 21 items. The experts were asked to rate the level of agreement with each item as assessment of medical professionalism on a 5-point scale scored as follows: 1 = Definitely include, 2 = Possibly include, 3 = Neutral, 4 = Possibly exclude, 5 = Definitely exclude. The expert was also invited to provide any comment on each item.

### Round 2

The authors reviewed the consensus rating and feedback from Round 1. Respondents to round 1 were contacted and provided with the group and individual ratings from round 1, as well as any feedback obtained from the experts. The experts were asked to re-rate the items with less than 70% agreement rate in round 1. Similar to round 1, a 5-point scale (1 = Definitely include, 2 = Possibly include, 3 = Neutral, 4 = Possibly exclude, 5 = Definitely exclude) was used. The expert was also invited to provide any comment on each item.

### Analysis

Consensus was deemed to be achieved when 70% of the experts chose to include (definitely include and possibly include) or exclude (definitely exclude and possibly exclude) the item. There is no universally accepted threshold for defining consensus as part of the Delphi process, with thresholds for consensus ranging from 55 to 100% in the published literature [31]. A predefined consensus level is an indicator of good quality Delphi research [32] and the consensus level is influenced by the study aims [33]. A 70% threshold was considered appropriate for this study and is consistent with other research using a modified Delphi technique [34, 35]. It was decided a priori that items with no consensus in the two rounds of Delphi would be included in the list of items to be used for pilot assessment of medical professionalism in residency program. Stability of responses was determined using Wilcoxon signed-rank test. Data were analyzed with Stata SE15.0 (Stata-Corp, College Station, TX, USA).

### Ethics

The SingHealth Centralized Institutional Review Board approved this study (Reference Number: 2016/3009). We obtained informed consent, which conformed to the principle outlined in the 1964 Declaration of Helsinki, from all the experts before the commencement of study.

## Results

**Fig. 2:**
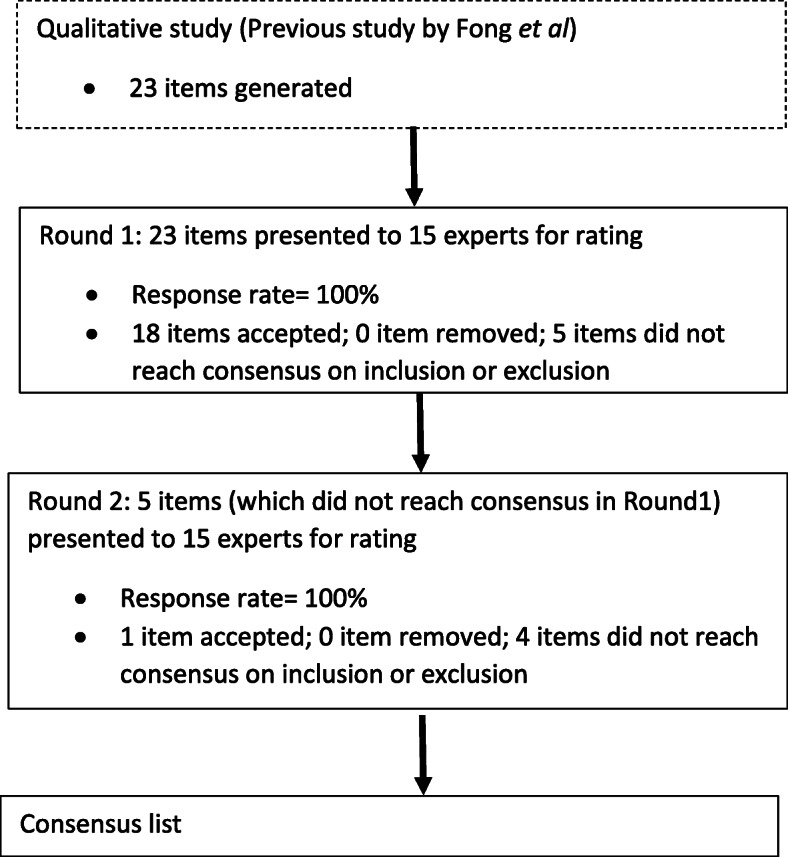
Delphi methodology and results

A total of 15 experts participated in this study (67% male, median age 45 years (37 to 66 years)), consisting of 11 program directors, three associate designated institutional officials (ADIO) and one designated institutional official (DIO). Around half of the faculty members came from medical disciplines, the rest spread across a wide spectrum of disciplines (surgical, diagnostic radiology, nuclear medicine and pathology, emergency medicine, pediatrics) (Table 1). All 15 experts participated in both rounds (response rate = 100%). Table 2 and Fig. 2 illustrate the summary of the results.

**Table 1:** Demographics of experts who participated in the Delphi survey

Characteristics	Median (Range) or Number (%)
Age, median (range)	45 (37–66)
Chinese, n (%)	13 (87)
Male, n (%)	10 (67)
Years as faculty, median (range)	12 (4–40)
Disciplines, n (%)	
Medical disciplines	7 (47)
Surgery	5 (33)
Emergency medicine	1 (7)
Radiology, nuclear medicine, pathology	1 (7)
Paediatrics	1 (7)

**Table 2:** Summary of results from rounds 1 and 2 of the Delphi survey

	Total number of items for scoring	Statements that reached consensus (≥ 70%) and were accepted	Statements that reached consensus (≥ 70%) and were removed
Round 1	23	18	0
Round 2	5	1	0

### Round 1

Completed questionnaires were received from all 15 experts. In round 1, 18 items (78%) achieved consensus for inclusion into the modified P-MEX tool. They included: listened actively to patient, showed interest in patient as a person, recognized and met patient needs, ensured continuity of patient care, maintained appropriate boundaries, communicated effectively with patient, demonstrated awareness of limitations, admitted errors or omissions, accepted feedback, maintained composure, was on time, completed tasks in a reliable fashion, was available to colleagues, maintained appropriate appearance, demonstrated respect for colleagues, avoided derogatory language, maintained patient confidentiality, demonstrated collegiality. The five remaining items (solicited feedback, advocated on behalf of a patient, extended his/herself to meet patient needs, used health resources appropriately, addressed own gaps in knowledge and skills) did not reach consensus for either inclusion or exclusion (Table 3).

**Table 3:** Results from Round 1 of the Delphi survey

Item	Percentage of experts who have chosen the category		
	Include	Neutral	Exclude
**Domain: Doctor-patient relationship skills**			
Listened actively to patients	93	7	0
Showed interest in patients as a person	73	0	27
Recognized and met patient needs	87	0	13
Extended his/herself to meet patient needs	27	13	60
Ensured continuity of patient care	87	7	7
Advocated on behalf of a patient	60	13	27
Maintained appropriate boundaries	100	0	0
Communicated effectively with patient	87	7	7
**Domain: Reflective skills**			
Demonstrated awareness of limitations	93	7	0
Admitted errors/omissions	100	0	0
Solicited feedback	27	33	40
Accepted feedback	100	0	0
Maintained composure in a difficult situation	100	0	0
**Domain: Time management**			
Was on time	87	7	7
Completed tasks in a reliable fashion	100	0	0
Was available to colleagues	73	13	13
**Domain: Inter-professional relationship skills**			
Maintained appropriate appearance	80	13	7
Addressed own gaps in knowledge and skills	60	13	27
Demonstrated respect for colleagues	93	7	0
Avoided derogatory language	93	7	0
Maintained patient confidentiality	100	0	0
Used health resources appropriately	47	20	33
Demonstrated collegiality	100	0	0

### Round 2

Completed questionnaires were received from all 15 experts. In round 2, 1 (4%) item (addressed own gaps in knowledge and skills) achieved consensus to be included. The 4 remaining items (solicited feedback, advocated on behalf of a patient, extended his/herself to meet patient needs, used health resources appropriately) did not reach consensus for either inclusion or exclusion (Table 4).

**Table 4:** Results from Round 2 of the Delphi survey

Item	Percentage of experts who have chosen the category		
	Include	Neutral	Exclude
**Domain: Doctor-patient relationship skills**			
Extended his/herself to meet patient needs	33	0	67
Advocated on behalf of a patient	60	27	13
**Domain: Reflective skills**			
Solicited feedback	27	27	47
**Domain: Inter-professional relationship skills**			
Addressed own gaps in knowledge and skills	93	7	0
Used health resources appropriately	53	7	40

### Consensus

A total of 19 items from the two rounds of the Delphi survey covering four domains of medical professionalism (Doctor-patient relationship skills, Reflective skills, Time management and Inter-professional relationship skills) were selected from the two-rounds of Delphi survey. Four items did not achieve consensus, and stability was observed for these items (Supplementary Table 1) [36]. Further rounds of Delphi were terminated as decided a priori.

## Discussion

Professionalism refers to a “set of values, behaviours and relationships that underpins the trust that the public has in doctors” [37] and there are often differences in how professionalism is defined and observed in different countries and cultures [38]. In Singapore, our residents comprising multi-ethnic groups often start their training of medical professionalism in medical school, where foundational platforms of medical professionalism included clinical competence, capacity to address ethical and legal issues and effective communication [37, 39], with virtues of medical professionalism such as honesty and integrity, responsibility and participation, respect and sensitivity, compassion and empathy taken as the foundational values of medical professionalism [40]. Thus, it is expected that these values continue to be reflected in their clinical practice upon graduation. In our study, we were able to achieve consensus on 19 items covering four domains of medical professionalism (Doctor-patient relationship skills, Reflective skills, Time management and Inter-professional relationship skills). All the 19 items, inclusive of the two new items of ‘communicated effectively with patient’ and ‘demonstrated collegiality’, were aligned with the aforementioned aspirational values that identified medical professionalism in our multi-ethnic society. In particular, our findings underscore the importance of recognising acceptable behaviours according to social, religious and cultural norms in a multi-ethnic and multi-cultural country such as Singapore. For example, the item on maintaining appropriate boundaries received 100% agreement for inclusion into the assessment of medical professionalism. However, as the P-MEX was primarily designed to assess observable behaviours for use in the clinical setting, only 24 of the 142 behaviours were eventually chosen to be evaluated [17], and this has also resulted in the P-MEX having a higher utility score when used in the clinical setting to assess trainees [13]. Thus, for practical reasons, it will not be able to assess all domains of professionalism completely e.g. altruism, excellence and humanism [39, 41]. In Korea, a previous study had identified eight categories of unethical and unprofessional behaviors- (a) substandard practice, (b) violation of work ethics, (c) misconduct related to conflict of interest, (d) dishonesty with patients, (e) violation of patient confidentiality, (f) lack of respect for patients, (g) lack of respect for colleagues, and (h) misconduct in research [42], of which six categories of unprofessional behaviour seem to mirror the 19 items found in our study.

In our study, 4 items “solicited feedback”, “advocated on behalf of a patient”, “extended his/herself to meet patient needs” and “used health resources appropriately” did not achieve the level of agreement to be included for both rounds, suggesting that these items may be less relevant in the local context as compared to other items in the list. This is largely in congruence with the findings of our previous qualitative study which showed that these items were less relevant to patients and/or faculty [29]*.* Similarly, in a study to define professionalism in anaesthesiology, “resourcefulness” which is similar to “used health resources appropriately”, was deemed to be less important [43]. This may be because of the difficulty to agree as to what is “appropriate use of health resources”, as raised by participants in this Delphi survey and in our previous qualitative study [29]. Modifications to these items may be needed in the future after the pilot testing of the modified P-MEX in our local context.

In our study, the two new items derived from the previous qualitative study- ‘communicated effectively with patient’ and ‘demonstrated collegiality’ both achieved the consensus to be included. This reflects the importance of effective communication and collaborative practice in medical professionalism locally, which is corroborated by the development and incorporation of various communication and interprofessional courses in local undergraduate and postgraduate medical education [44–47]. Effective communication with patients and other healthcare personnel, as well as respect for patients and colleagues were recognized as increasingly important domains of medical professionalism that need additional attention and nurturing [48]. The emphasis on collegiality may also reflect the collectivist nature of Asian culture based on the Hofstede’s cultural dimension theory [27, 42] and the increasing recognition that medical professionals practice in a community of practice [49]. Other Asian countries exploring the use of the P-MEX should consider including “communicated effectively with patient” and “demonstrated collegiality” to better reflect the medical professionalism construct.

Key strengths of this study include involvement of senior faculty, including the DIO and ADIO, who are involved in the disciplinary committees handling issues of lapses in professionalism in the residency programs. In addition, faculty from a range of disciplines (both surgical and medical specialties) participated in the Delphi survey and of different ethnicities. There was also no expert drop-out in the two rounds of Delphi survey.

Limitations of this study include the arbitrariness of the cut-off point utilised. The cut-off point of 70% adopted in our study was widely used in other studies [34, 35]. However, at this threshold, our findings should be taken as the best achievable consensus given the lack of robust evidence in this field rather than as evidence of absolute unanimity. In addition, the study may have selection bias as the experts were selected by the researchers. However, considerable care was taken during the study to select the experts based on their experience in handling of disciplinary issues in the residency programs, and to include faculty of various years of experience, gender, ethnicity and across various surgical and medical disciplines. Moreover, the framework of medical professionalism in the P-MEX containing 4 domains - Doctor-patient relationship, Reflective, Time management, and Inter professional relationship - may not cover entire continuum of professionalism. Therefore, we may have missed certain attributes that define medical professionalism such as social accountability and altruism. Nevertheless, our qualitative [29] and survey studies (under review) with patients and faculty members showed that the four domains of P-MEX broadly covered medical professionalism in our setting with an addition of 2 new sub-domains- (1) communicated effectively with patient and (2) demonstrated collegiality.

## Conclusions

A list of 19 items covering four domains of medical professionalism (Doctor-patient relationship skills, Reflective skills, Time management and Inter-professional relationship skills) of the P-MEX was obtained from the Delphi study. The 4 other items (solicited feedback, advocated on behalf of a patient, extended his/herself to meet patient needs, used health resources appropriately) did not reach consensus for either inclusion or exclusion. The findings of this study can provide guidance for faculty and institutions who want to introduce assessment of medical professionalism in the curriculum of medical trainees. In particular, other Asian countries exploring the use of the P-MEX may consider incorporating items such as “communicated effectively with patient” and “demonstrated collegiality” to have a more comprehensive and culturally pertinent assessment of the medical professionalism.

## Supplementary information

**Additional file 1: Supplementary table 1.** Wilcoxon signed-rank test results comparing responses from round 1 and round 2 of the Delphi survey.

## Data Availability

The datasets used and/or analysed during the current study are available from the corresponding author on reasonable request.

## Abbreviations

ADIO: Associate designated institutional officials
DIO: Designated institutional official
P-MEX: Professionalism Mini Evaluation Exercise
