# Supplementary material for: Assessment of medical professionalism using the Professionalism Mini Evaluation Exercise (P-MEX) in a multi-ethnic society: a Delphi study
Source: BMC Med Educ. 2020 Jul 14;20:225. doi: 10.1186/s12909-020-02147-9 (PMC7362541; doi:10.1186/s12909-020-02147-9)
Supplement: Supplementary file 1 — Additional file 1: Supplementary table 1. Wilcoxon signed-rank test results comparing responses from round 1 and round 2 of the Delphi survey. [file 12909_2020_2147_MOESM1_ESM.docx]

Supplementary table 1: Wilcoxon signed-rank test results comparing responses from round 1 and round 2 of the Delphi survey

| Item | Z-score | Significance |
| --- | --- | --- |
| Extended his/herself to meet patient needs | 0.586 | 0.558 |
| Advocated on behalf of a patient | -0.757 | 0.449 |
| Solicited feedback | 0.887 | 0.375 |
| Addressed own gaps in knowledge and skills | -1.776 | 0.076 |
| Used health resources appropriately | -0.296 | 0.767 |
